# Probiotics and mediterranean diet for breast cancer management and prevention?

**DOI:** 10.15698/cst2025.05.303

**Published:** 2025-05-08

**Authors:** Ehssan A. Sharif-Askari, Khadija M. Atoui, Ali K. Mteyrek, Lama M. Fawaz

**Affiliations:** 1 Biomedical Science Department, School of Arts & Sciences, Lebanese International University, Tyre, Lebanon.; 2 Department of Biological and Chemical Sciences, School of Arts and Sciences, Lebanese International University, Tyre, Lebanon.; 3 Neuroimmunology Unit, Montreal Neurological Institute, McGill University, Quebec, Canada.

**Keywords:** gut microbiota, cancer, mediterranean diet, anticarcinogenic activities, personalized dietary strategies, cancer prevention

## Abstract

The human gut microbiota, a diverse community of beneficial normal flora microorganisms, significantly influences physiological function and the immune response. Various microbiota strains have shown promise in supporting clinical treatment of chronic diseases, including cancer, by potentially providing antioxidative and anti-tumorigenic effects in both *in vivo* and *in vitro* studies. Breast cancer, which ranks amongst the top five cancer types common worldwide and particularly in Mediterranean countries, has been showing high incidence and prevalence. In breast cancer, microbiota composition, hormonal dynamics, and dietary choices are believed to play significant roles. Hence, the Mediterranean diet, known for its microbiota-friendly features, emerges as a potential protective factor against breast cancer development, highlighting the potential for personalized dietary strategies in cancer prevention. This comprehensive review highlights the emerging mechanisms by which probiotics support our immune system during different physiological activities. It also discusses their potential role, along with nutrition intervention, in improving essential clinical treatment outcomes in breast cancer patients and survivors, suggesting potential supportive strategies that go hand in hand with clinical strategies. Unfortunately, very little research addresses the possible clinical implications of probiotics and dietary habits on breast cancer, despite the promising results, calling for further studies and actions.

## Abbreviations

BC - breast cancer,

EMR - East Mediterranean Regions,

ER - estrogen receptor,

FM - fermented milk,

GI - gastrointestinal,

LP-CLA - L. plantarum - Conjugated Linoleic Acid,

MD - Mediterranean diet,

miRNA - microRNA,

ROS - reactive oxygen species,

SeNp - Selenium nanoparticles,

TLR - Toll-like receptor,

UPF - ultra-processed food.

## BACKGROUND

Breast cancer (BC) is rising in both prevalence and incidence worldwide, and due to the scarcity of research, it has become imperative to shed light on this rapidly growing disease. Studies have found a clear correlation between BC progression and the gut microbiome, which has opened avenues for new research, particularly discussing the role of probiotics in cancer prevention, support of medical treatment and, possibly, treatment. Healthy dietary patterns, as in ones high in micronutrients, bioactive compounds, and probiotics, like the Mediterranean diet, have been proven to improve clinical and medical treatment outcomes in BC patients. These active ingredients tend to enhance the immune response and reduce factors contributing to cancer progression, such as oxidative stress and inflammation.

## INTRODUCTION

BC is one of the high-ranking types of cancer on an international scale. It is characterized by its clinical heterogeneity and complexity, which makes it a leading global health concern, mainly in women. The potentially lethal disease begins with abnormal growth of breast cells in the milk ducts and/or the milk-producing lobules, forming tumors that may spread throughout the body and become fatal 1. Regardless of the major efforts at early detection and new treatments, there are more than 2.3 million cases of BC that occur each year, and it is the leading cause of female cancer death in 95% of countries 2. In East Mediterranean Regions (EMR), BC was the leading cancer in females in 2022 with 32.6% of new cases and the first-ranking in types of cancers causing deaths with 10.9% 3. However, due to increased public health awareness, early detection, and advanced cancer therapies, the survival rate among BC patients is rising 4. Moreover, a wide number of research focusing on various dietary patterns, micronutrients and bioactive compounds, indicates the importance of nutrition intervention in improving treatment outcomes in BC patients and survivors 5.

The human gastrointestinal (GI) tractis colonized by a diversity of bacteria, viruses, archaea, and unicellular eukaryotes, termed as "gut microbiota" 6,7,8, which is identified as a vital organ forming its multipronged networking with other organs 8. Although all human body surfaces are colonized by microbes, a compelling number of microbes resides in the GI tract/gut 8. The intestinal microbiota encompasses more than 1500 species, distributed in more than 50 different phyla 9, where *Bacteroidetes* and *Firmicutes* followed by *Proteobacteria*, *Fusobacteria*,* Tenericutes*, *Actinobacteria* and *Verrucomicrobia* are the most dominant phyla, making up to 90% of the total microbial population in humans 10. The microbiota proposes numerous advantages to the host, by providing a range of physiological functions such as enhancing gut integrity or influencing the intestinal epithelium 11 as well as controlling epithelial cell proliferation and differentiation 12. They play a central role in guarding host health in both healthy and diseased states. Their function is mainly characterized by immunoregulation and maintenance of homeostasis.

The term *probiotics* has been widely used for several years, where Nobel Prize laureate Elie Metchnikoff presupposed their immunomodulatory effects 13. They are defined as "live microorganisms that, when administered in adequate amounts, confer a health benefit on the host" 14. They can modulate the intestinal immunity and they are able to restore a balanced gut microbiome introducing beneficial function to the gut microbial community. Moreover, they are able to enhance intestinal homeostasis and strengthen intestinal barrier function. Due to their potential benefit in cancer prevention and as adjunctive therapy during anticancer treatments, probiotics have gained a significant attention in the scientific community 15,16,17,18,19,20. According to a recent report by Kaufmann *et al*., the intake of probiotics and prebiotics, nondigestible food components that serve as food for host gut microbiota, prevented chemotherapy-induced diarrhea and microbiota imbalances during BC treatment 21. In fact, the potential of probiotics extends to inhibiting BC progression and is characterized by the prevention of pathogen adhesion to mucus layer, reduction of nitric oxide production, prevention of cancer cell propagation, as well as modulation of certain cytokine production and promoting cell proliferation, as evidenced by animal models and cell-based experiments 22,23,24. One interesting species of probiotics is *Lactobacilli,* which plays a crucial role in different aspects of overall human health regulation and immune modulation. For instance, oral administration of *Lactobacillus acidophilus *and Cyclophosphamide (a cytostatic drug used for BC therapy) significantly reduced tumor growth in 4T1 tumor-bearing mice (P = 0.00), enhanced lymphocyte proliferation and altered cytokine production. This alteration is characterized by increasing IFN-γ and decreasing IL-4 and TGF-β (P < 0.05), thereby promoting an anti-tumor Th1 immune response 22. **Table 1** summarizes probiotics contributions in human overall health and cancer treatment.

**Table 1:** Summery of probiotics contributions in human overall health and cancer treatment. * Lactobacillus rhamnosus, *Saccharomyces cerevisiae var. boulardii*, *Bifidobacterium lactis* HN019, *Lactobacillus rhamnosus* HN001, a probiotic blend containing unspecified strains of *Saccharomyces cerevisiae var. boulardii*, *Lactobacillus acidophilus* NCFM, and *Bifidobacterium lactis*.

**Probiotic**	**Effect**	**Reference**
Probiotic mixture*	Mitigate the after-chemotherapy symptoms such as irritable bowel syndrome, diarrhea, or constipation	21
*Lactobacillus acidophilus*	Reduced tumor growth in 4T1, enhanced Th1 lymphocyte proliferation, increased IFN-γ and decreased IL-4 and TGF-β	22
*Lactobacillus rhamnosus Lcr35*	change the expressions of genes, involved in immune responses in Monocyte derived dendritic cells	26
	Increase the production of: TNF, IL-1β, IL-12p70, IL-12p40, and IL-23	
	Upregulation of CD86, CD83, HLA-DR, and TLR4 and Downregulation of DC-SIGN, MR, and CD14	
*Lactobacillus casei CRL* 431	Decrease of tumor volume and angiogenesis, with change in cytokine profile especially IL-6	27
*Lacticaseibacillus paracasei SD1*, *L.* *rhamnosus* SD4 and SD11, and L*imosilactobacillus* *fermentum* SD7	Induce human β-defensin 2 and 4, IL-1β, IL-6, IL-8, and TNF-α expressions in human gingival epithelial cells	28 29 30
*Lactobacillus casei*, *L.* *acidophilus*, *L.* *rhamnosus*, *L.* *delbrueckii *subsp. *bulgaricus*, *L. plantarum, L. lactis, Streptococcus thermophilus*	Increase the number of intestinal IgA-producing cells	31
*Lactobacillus casei* *CRL 431* and L*actobacillus helveticus R389*	Increase IL-6 levels secreted in a TLR2-dependent manner which triggers the B cell clonal expansion and rises of intestinal IgA-producing cell number	32
*Bifidobacterium breve* *IPLA 20004* and *bifidum* *LMG13195*	Treg cell differentiation and the release of CCL20, CCL22, CXCL10, and CXCL11	33
*Bifidobacterium lactis sp*.*420*	Modulates expression of cyclooxygenase, controlling the potential anti-inflammatory and anticarcinogenic properties of cancer	42
*Clostridia *clusters IV and XIVa	Induce a local and systemic Treg cell response	43
L. paracasei EPS DA-BACS	Decrease NO production by LPS-activated RAW 264.7 cells	44
*L. reuteri*	Induce anti-inflammatory CD4+ CD25+ Treg cells	45
*Lactobacillus* and *Bifidobacterium*	Decrease oxidative radicals’ secretion by enhancing antioxidants enzymes	52
*Lactobacilli*	Chelating Fe^2+ ^and Cu^2+ ^ions released by ROS	57
	Inhibit the activity of NADPH oxidase Inhibit expression of NOX-1 and NOX-4 mRNA	58
	Interleukin-8 production by short interfering RNA directed against TLR5	96
*Leuconostoc mesenteroides*	Downregulate mir-21 and mir-200b, inducing in apoptosis in colon cancer by upregulating MAPK1, Bax, and caspase-3 and downregulating AKT, NF-κB, and Bcl-XL	64
*L. acidophilus* ATCC strain 4356	Upregulation of mir-21 and the downregulation of mir-155, resulting in a reduction of apoptosis, necrosis, and inflammation	65
*L. fermentum* CECT5716 and *L. salivarius* CECT5713	Reduce the expression of the pro-inflammatory cytokine IL-1β in mice by downregulating miR-155, miR-223, and miR-150 and upregulating miR-143	66
*Lactobacillus *and *Streptococcus*	Generation of NK cells	73
*Streptococcus thermophilus*	Antioxidant production, which mitigates DNA damage and neutralizes ROS	73
*LGG, L. rhamnosus KLDS, L. helveticus IMAU70129,* and *L. casei IMAU60214*	Modulate pro-inflammatory macrophages by synthesizing pro-inflammatory cytokines, signaling cascades such as the NF-κb and TLR2 pathways, as well as ROS	94
*L. plantarum and L. brevis*	Elevated IFN-γ in both strains and IL-2 in *L.plantarum*, enhanced NK cells activity in both strains	100 101
*L. plantarum* – Conjugated Linoleic Acid	Notable elevation in the pro-apoptotic protein Bax	102
*L. casei *Shirota	Decreased BC risk in Japanese women	104

In EMR, the exact prevalence and incidence of BC remains vague due to the lack of structured cancer registries. In addition, data on disease mortality is also absent and largely unknown. For example, according to the Lebanese Ministry of Public Health, the incidence of BC in 2016 was nearly double the global average, with 96.8 cases per 100,000 women across all age groups 25. Despite having the advantage of Mediterranean Diet (MD) in the region, BC has been increasing in both prevalence and incidence in EMR, posing an important question about the actual reason of this rise.

In this review, we will discuss the potential therapeutic benefits for using probiotics and describe their advantageous effects along with adopting healthy dietary patterns, such as the MD, to support medical interventions in BC. Lastly, we will highlight potential factors that may counteract the beneficial effects of probiotics and MD and contribute to the increased prevalence of BC in EMR.

## MECHANISTIC OVERVIEW OF PROBIOTICS EFFECTS ON HEALTH

### Probiotics and immune regulation 

**Figure 1.**
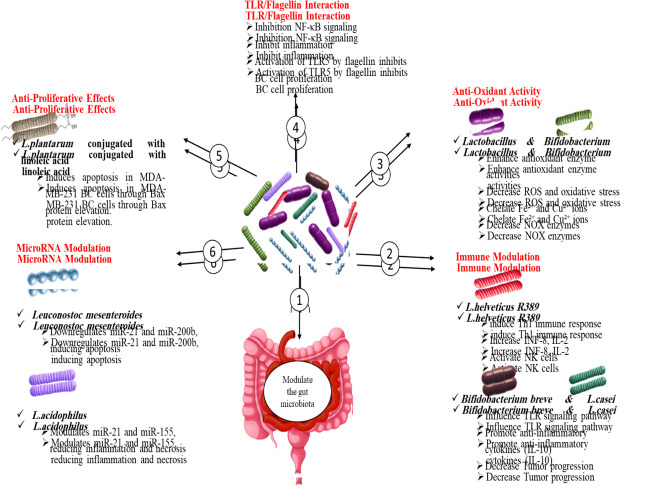
FIGURE 1: The beneficial effects of probiotics on gut microbiota and host health. This figure illustrates how probiotics influence various physiological processes. (1) Probiotics modulate the gut microbiota, promoting a balanced microbial ecosystem. (2) They contribute to immune modulation by interacting with host immune cells and enhancing immune responses. (3) Certain probiotic strains exhibit anti-oxidant activity, reducing oxidative stress and protecting cells from damage. (4) Probiotics engage in TLR/Flagellin interactions, influencing immune signaling and inflammation pathways. (5) Some probiotics demonstrate anti-proliferative effects, which may help in inhibiting the growth of harmful or cancerous cells. (6) MicroRNA modulation by probiotics affects gene expression, contributing to various biological processes and overall health benefits.

Probiotics in the human intestine regularly communicate with immune cells. They regulate their action and help maintain homeostasis (**Figure 1**). These microbiotas regulate the immune response not only by direct contact with immune cells, but also by modulating secretion of cytokines and controlling membrane protein expression and gene expression. In fact, high treatment ‘doses’ of *Lactobacillus rhamnosus* *Lcr35* (multiplicity of infection MOI 100 compared to MOI 0.01) were incrementally shown to induce a large-scale change in gene expression, specifically involving immune responses in human monocyte-derived immature dendritic cells. They also induced a strong dose-dependent increase of the production of the pro-Th1/Th17 cytokines, such as TNF, IL-1β, IL-12p70, IL-12p40, and IL-23, compared to a slight increase in IL-10 production 26. Moreover, it can stimulate a dose-dependent maturation of the dendritic cell membrane phenotype, *in vitro*, with an upregulation of the membrane expression of CD86, CD83, HLA-DR, and TLR4, and a downregulation of DC-SIGN, mannose receptor (MR), and CD14 26.

To emphasize the role of probiotics in the maintenance of anti-tumor-IL environment in BC, an experiment was conduct-ed, when *Lactobacillus casei CRL* *431* fermented milk (FM) was administered to tumor-bearing mice where the tumors reached a certain volume against control and milk-administrated mice. This administration decreased tumor volume and increased mice survival, where the effect was related to a decrease of tumor angiogenesis and changes in the cytokine profile in blood serum, especially IL-6 which is mainly related to the angiogenesis induction 27. Mice treated with FM showed significantly reduced blood vessel area in tumors and IL-6 levels (P < 0.05), alongside the highest survival rate (50% vs. 25-28.5% in control/milk groups) and increased CD4/CD8 double-positive and CD4+ cells in tumors (P < 0.05) compared to control and milk groups 27.

A recent meta-analysis suggested that the local administration of probiotics such as *L. casei Shirota, Lactobacillus fermentum *and* L. rhamnosus, *as well as* Bifidobacterium animalis, *might influence the release of some salivary cytokines. It demonstrated that L*acticaseibacillus paracasei SD1*, *L*. *rhamnosus* *SD4* and *SD11*, and *Limosilactobacillus* *fermentum* *SD7* induced human β-defensin 2 and 4, IL-1β, IL-6, IL-8, and TNF-α expressions in human gingival epithelial cells 28,29,30.

On the other hand, studies proved that oral administration of probiotics, especially *Lactobacilli species *will influence the IgA secretion. For instance, the oral administration of *L. casei*, *L.* *acidophilus*, L. *rhamnosus*, *Lactobacillus* *delbrueckii *subsp. *bulgaricus*, *Lactobacillus* *plantarum* and *Lactobacillus* *lactis*, as well as *Streptococcus thermophilus*, was reported to increase the number of intestinal IgA-producing cells in a dose-dependent manner 31. In another study, after oral administration of *L. casei* *CRL 431* and *Lactobacillus helveticus R389*, the increased IL-6 levels secreted in a TLR2-dependent manner were described to cause the rise of intestinal IgA-producing cell number without a simultaneous CD4+ T-cell number increase 32. These results suggest that *Lactobacilli *can trigger the B cell clonal expansion through IL-6 production in order to release IgAs.

Interestingly, probiotics can also effectively regulate chemokine secretion. *Bifidobacterium breve* *IPLA 20004* and *bifidum* *LMG13195 *favored Treg cell differentiation and the release of CCL20, CCL22, CXCL10, and CXCL11, capable of recruiting effector immunoreactive lymphocytes 33. Although these studies indicate the favored release of various classes of both cytokines and chemokines after probiotic administration, the molecular mechanisms of these immunological processes remain unknown.

It is also important to mention that some recent literature denotes that the gut microbiota influences the immune system response to vaccination. Authors have investigated the effect of probiotics on immunization after influenza vaccination and addressed a significant improvement in the H1N1, H3N2 and B strain serum-protection rate in adults, suggesting that probiotics are influencing seroconversion in adults vaccinated for influenza, thus increasing immunogenicity 34. However, other studies did not show any effect or inverse effects on this topic, calling for further research in this field 35,36.

### Probiotics and cancer inflammation

Inflammation is often associated with the progression and development of cancer; additionally, it is considered one of the main cancer hallmarks. For the past decades, researchers have been delving into the potential of targeting inflammation in cancer treatment. Most evidence supports the exacerbating effect of inflammation in cancer 37, as approximately 15-20% of all infection, chronic inflammation, or autoimmunity cases are followed by cancer development in the same tissue or organ 38,39.

Specifically, evidence suggests that acute inflammation promotes cancer progression and metastasis. Moreover, if the acute inflammatory reaction is not resolved in time, it will cause chronic inflammation, ultimately leading to an immunosuppressive microenvironment with a large number of immunosuppressive cells (M2 macrophages, Myeloid-derived Suppressor Cells (MDSCs), T reg cells, etc.) and cytokines 39,40. Subsequently, these changes will promote the activation of oncogenes, DNA and protein damage, release of Reactive Oxygen Species (ROS), and influence multiple signaling pathways, including NF-κB, K-RAS, and P53, eventually leading to cancer and other chronic diseases 40.

Furthermore, inflammation-related signaling undermines the process of eliminating potentially cancerous cells, thereby allowing cells with pro-oncogenic changes to persist and progress toward tumor formation 41. Therefore, reducing inflammation may be a promising key in preventing tumor formation, and, interestingly, some probiotics play a key role in promoting anti-inflammatory processes. For example, *Bifidobacterium lactis* *sp.420* influences the host’s expression of cyclooxygenase, which plays a role in regulating its potential anti-inflammatory and anticarcinogenic effects in cancer 42. Moreover, many bacteria are able to control Treg maturation, stimulate the anti-inflammatory fork of the adaptive immune system, or drive IL-10 production. For instance, it has been demonstrated that a mix of 46 strains of *Clostridia *clusters IV and XIVa can induce a local and systemic Treg cellular response in mice 43.

According to a recent study, Exopolysaccharide-Producing *L. paracasei *(*L. paracasei EPS DA-BACS*) exhibited anti-inflammatory activities, as heat-treated *L. paracasei EPS DA-BACS* decreased the secretion of NO by LPS-activated RAW 264.7 macrophage cells 44. In addition, another study demonstrated that *Lactobacillus reuteri* protected against BC by inducing anti-inflammatory CD4+ CD25+ Treg cells, which in turn led to breast hyperplasia and an elevation in pretumor lesions 45.

Currently, probiotics are believed to enhance the immunologic barrier through inflammatory responses characterized by augmentation of intestinal IgA 46,47,48. The secretory IgA antibodies in the gut are part of the common mucosal immune system, which consists of the respiratory tract, lacrimal, salivary, and mammary glands. Consequently**,** the immune responses at these mucosal surfaces will be affected by any initiated immune response in the gut-associated lymphoid tissue 49.

These findings demonstrate the significant role of inflammation in cancer progression and the potential role of probiotics in mitigating inflammation-driven tumor development. By supporting the immune system and creating an anti-inflammatory environment, probiotics may offer a natural way to aid in cancer prevention and treatment.

### Probiotics as antioxidants

Probiotics have strong redox systems and antioxidant properties, reducing ROS accumulation, which also can significantly contribute to inflammatory diseases, as well as aging and cancer 50. *Lactobacillus *and* Bifidobacterium* species exhibit remarkable antioxidant capabilities, offering a level of defense against oxidative stress 51. They contribute to enhancing the equilibrium between oxidative and antioxidant systems, ultimately reducing the generation of free radicals by bolstering the capacities of antioxidant enzymes 52.

In fact, *Lactobacilli* are aerotolerant anaerobes that do not use oxygen but fermentation in order to produce ATP 53,54. They are common probiotic bacteria found in yogurt and have diverse applications which help maintain human well-being, such as treating diarrhea, vaginal infections, and skin disorders 55. These species have demonstrated a significant scavenging capability against 2,2-diphenyl-1-picrylhydrazyl free radical molecule, O_2_^−^, and H_2_O_2_ *in vitro* 56. In addition to their nonenzymatic defense mechanism against oxidative stress, relying on the chelation of both Fe^2+^ and Cu^2+^, which are highly active ions produced by ROS 57. Furthermore, when different *Lactobacillus* strains are combined (with?), supplementation appears to reduce the activity of NADPH oxidase (NOX) and the expression of NOX-1 and NOX-4 mRNA, key contributors to ROS generation 58.

### Probiotics and microRNA regulation 

Recently, research has shifted to explore how microRNAs (miRNAs)—small noncoding RNA (ncRNA) molecules that are pivotal in gene expression regulation and implicated in cancer development—may serve as a link between the microbiota and host physiology by influencing gene expression in host cells 59. Notably, miRNAs can serve as biomarkers for the early detection and monitoring of cancer prognosis. For example, one study showed that certain miRNA species, such as miR-21, miR-106a, and miR-155, are up-regulated in tumor samples compared to non-tumor samples, whereas others, such as miR-126, miR-199a, and miR-335, are down-regulated in tumor samples 60. This highlights a correlation between BC and circulating miRNA levels.

Additionally, the expression levels of other miRNAs, including miR-21, miR-126, miR-155, miR-199a, and miR-335, are associated with clinicopathological characteristics, such as histologic tumor grades of BC and the expression of sex hormone receptors 60. In particular, miR-21 has been identified as an oncogene due to its tumor growth-promoting ability. By targeting tumor suppressor genes, TPM1 and PDCD4, and inhibiting their expression, miR-21’s overexpression is closely linked to BC occurrence and progression, making it a promising biomarker for the diagnosis and prognosis of the disease 61.

Interestingly, miRNA expression profiles vary between cancer types. For instance, metastatic luminal A BC exhibits elevated levels of miR-331, while miR-195 is downregulated 62. Moreover, the distinguishing feature between luminal A and luminal B cancers lies in the increased expression of the miRNA cluster miR-99a/let-7c/miR-125b in luminal A. This cluster is associated with improved overall survival in luminal A patients, particularly those with high miR-99a levels. Accordingly, this miRNA cluster serves as both a potential biomarker for differentiating luminal A from luminal B cancers and a prognostic indicator within the luminal A group, where low levels of these miRNAs are associated with poorer survival outcomes 63.

Furthermore, it is now well-known that miRNAs play a significant role in the genesis of neoplastic diseases, and their modulation represents an important therapeutic opportunity. Several studies have confirmed a close connection between microbiota and miRNAs. For example, *Leuconostoc mesenteroides* probiotics downregulate miR-21 and miR-200b, leading to apoptosis in colon cancer cell lines by upregulating MAPK1, Bax, and caspase-3 and downregulating AKT, NF-κB, and Bcl-XL 64. Conversely, *L. acidophilus* ATCC strain 4356 induces the upregulation of miR-21 and the downregulation of miR-155, resulting in a reduction of apoptosis, necrosis, and inflammation 65. Similarly, *L. fermentum* CECT5716 and *Lactobacillus salivarius* CECT5713 reduce the expression of the pro-inflammatory cytokine IL-1β in mice by downregulating miR-155, miR-223, and miR-150 and upregulating miR-143 66. Although the interplay between probiotics and miRNAs has been explored in some cancers, how probiotics modulate miRNAs in BC remains largely undetermined. This unexplored area of research holds significant promise, not only for advancing cancer treatments but also for developing innovative therapeutic strategies for other chronic diseases.

## PROBIOTICS AND BREAST CANCER DEFENSE

### Microbial environment fluctuation

The microbial community in human breast tissue is diverse and unique, differing significantly from other regions of the body 67. This distinctiveness holds true regardless of the specific sample site within the breast, the individual’s age, geographical location, or pregnancy history 67. The unique breast microbiota pattern consists, in decreasing order, of *Proteobacteria*, *Firmicutes*, *Actinobacteria*, and *Bacteroidetes* 67. Others reported a relative myriad of *Bacillus*, *Enterobacteriaceae* and *Staphylococcus* and a decline in other bacteria, including *Lactobacillus*, in BC when compared with the histologically normal tissue adjacent to the tumor 68,69. The composition and abundance of some specific bacterial taxa between BC patients and healthy individuals is shown in (**Table 2**).

**Table 2:** Difference in the microbiotic environment in breast tissue between Health individuals and breast cancer patients.

**Healthy breast tissue microbiota**	**References**
*Proteobacteria*	67
*Firmicutes*	67
*Actinobacteria*	67
*Bacteroidetes*	67
*Lactobacillus*	73
*Streptococcus*	73
*Sphingomonadaceae*	82
**Breast cancer tissue microbiota**	**References**
*Bacillus*	68 69
*Enterobacteriaceae*	68 69
*Staphylococcus*	68 69
*Alistipes*	82

Breast milk’s specific microbiota composition and the potential for bacteria to create a unique microbiome within the breast ducts, crossing through the nipple, present intriguing dimensions in understanding microbial influences on breast health 70. Examining fecal samples from BC patients unveils a positive correlation between the abundance of *Streptococcus*, part of the normal microbiota found in GI and other body sites, and the presence of β-glucuronidase and/or β-glucosidase enzymes 71. These enzymes play a major role in estrogen metabolism by cleaving glucuronidated estrogenic compounds, thereby reactivating estrogen, which can then be readily reabsorbed 72. These enzymes, facilitating estrogen recirculation, further emphasize the systemic impact of microbial modulation 71.

The elaborated connection between breast health and microbiota composition offers further insights into the potential of cancer prevention. The effects of some probiotics strains on BC are presented in **Table 3**. Notably, the microbiota of healthy breast tissue is dominated by* Lactobacillus *and *Streptococcus* which are recognized for their health-promoting nature. These bacteria possess important anticarcinogenic activities, involving the generation of NK cells that regulate tumor progression 73. In addition, *Streptococcus thermophilus* provides observed antioxidant production, which mitigates DNA damage and neutralizes ROS 73. To date, data representing correlation between probiotics and immune cells, especially NK cells, is quite insufficient. Therefore, advanced studies are still required to explore the fundamental mechanisms of these processes.

**Table 3:** Effect of some probiotics on breast cancer.. * Lactobacillus rhamnosus, *Saccharomyces cerevisiae var. boulardii*, *Bifidobacterium lactis* HN019, *Lactobacillus rhamnosus* HN001, a probiotic blend containing unspecified strains of *Saccharomyces cerevisiae var. boulardii*, *Lactobacillus acidophilus* NCFM, and *Bifidobacterium lactis*.

**Probiotic**	**Effect**	**Reference**
Probiotic mixture *	Mitigate the after-chemotherapy symptoms such as irritable bowel syndrome, diarrhea, or constipation	21
*Lactobacillus casei *CRL 431	Decrease tumor angiogenesis by controlling IL-6 levels	27
*Streptococcus*	Facilitate estrogen recirculation through increasing of β-glucuronidase and/or β-glucosidase enzymes	71
*Lactobacillus *and *Streptococcus*	Anticarcinogenic activities and generation of NK cells	73
*Streptococcus thermophilus*	Mitigates DNA damage and neutralizes reactive oxygen species (ROS)	73
*Lactobacillus plantarum*	Apoptosis induction through increasing Bax pro-apoptotic protein	102
*L. plantarum and L. brevis*	Elevated IFN-γ in both strains and IL-2 in *L.plantarum*, enhanced NK cells activity in both strains	100 101
*L. casei *Shirota	Decreased BC risk in Japanese women	104

### Probiotics and hormonal regulation 

The link between sex hormone dysregulation and BC development is a well-established risk factor, both clinically and molecularly in several BC subtypes 74. It has been demonstrated that a subset of microbes within the GI tract plays a crucial role in influencing estrogen metabolism and regulating the equilibrium of circulating and excreted hormone levels 75. Estrogen binds to Estrogen Receptor (ER) to activate signaling pathways and exert biological properties such as stimulating cellular proliferation and inhibiting apoptosis 76. ER*α* is the leading ER subtype in the mammary epithelium and performs a key role in mammary gland biology and in BC progression 77,78. Upon the binding of estrogen to ER*α*, the ligand activated ER*α* translocates to the nucleus, binds to the responsive element in the target gene promoter, and stimulates gene transcription (genomic/nuclear signaling) 79,80. One of these genes is *Bcl-2* which inhibits apoptosis of BC cells 81.

Studying this connection, there is a proposed association between estrogen conjugation by β-glucuronidase and microbiota dysbiosis in women with BC 82. Higher levels of this enzyme in nipple aspirate fluid from BC patients, particularly with an abundance of *Alistipes*, highlight potential microbial contributions to hormonal dynamics in cancer 82. As a matter of fact, an unclassified genus of the *Sphingomonadaceae* family was observed in healthy women 82. A study of Xuan *et al.*, showed that the *Sphingomonas Yanoidkuyae* species were more abundant in healthy breast tissues. This genus of *Sphingomonas* is a member of the *Sphingomonadaceae* family known for their capacity to degrade aromatic hydrocarbons and polycyclic aromatic hydrocarbons 83,84. *Sphingomonas Yanoidkuyae* was found to be able to degrade estrogen 84, which may infer its potential benefit in therapy, especially for estrogen-involved BC.

### Probiotics and flagellin/TLR interaction in cancer

The effect of probiotics extends to show a modulation and activation role of Toll-like receptors (TLRs), thus suggestiing an additional mechanism through which they can contribute to cancer prevention. TLRs are highly conserved pattern-recognition receptors, mainly expressed in human epithelial and immune cells, which play a crucial role in innate immunity 85,86. They primarily induce the synthesis and release of inflammatory cytokines and chemokines, thereby initiating the inflammatory response 87,88. Recently, evidence has suggested that probiotics modulate the immune system by interacting with TLRs, leading to the production of anti-inflammatory cytokines, such as TNF in epithelial cells, inhibition of NF-κB in macrophages, and the production of IL-8, which is required to recruit neutrophils 89.

Many probiotics secrete a byproduct called short-chain fatty acids (SCFAs) 90. Studies have shown that SCFAs can reduce the production of cytokines such as TNF-α, and IL-12, while simultaneously promoting the production of anti-inflammatory cytokines like IL-10 91. They also reduce the infiltration of leukocytes into colonic mucosa and directly suppress the immune response by inhibiting TLR-4 receptor signaling 92. Recently, a study with the *L. casei* strain demonstrated that probiotics induce macrophage polarization through the TLR4-mediated pathway, inhibiting cancer progression 93. Furthermore, *LGG, L. rhamnosus KLDS, L. helveticus IMAU70129,* and *L. casei IMAU60214* modulate pro-inflammatory macrophages by synthesizing pro-inflammatory mediators, including cytokines, signaling cascades such as the NF-κB and TLR2 pathways, as well as ROS 94.

Further studies suggest that TLR expression can serve as a biomarker of cancer pathogenesis and progression. Chronic activation of TLRs is implicated in BC initiation and promotion. TLR5 receptors, which are highly expressed in breast carcinomas, exhibit potent anti-tumor activity when activated by the flagellin ligand, offering a potential avenue for therapeutic intervention 95. Flagellin, used by bacteria for motility, is present in many probiotics 96,97. *Lactobacilli* strains induce IL-8 production in cultured human intestinal epithelial cells in a manner suppressed by short interfering RNA directed against TLR5 96. To investigate the effect of flagellin/TLR5 signaling on tumor cell proliferation, Cai *et al*., performed a BrdUrd incorporation assay to track DNA synthesis as an indicator of cell proliferation. MCF-7 cells, which are human BC cells lined with estrogen, progesterone, and glucocorticoid receptors, were treated with flagellin, and the number of BrdUrd-incorporated cells was determined daily by flow cytometry 98. A decline in the percentage of BrdUrd-incorporated cells from 46% to 32% was detected after one day of flagellin treatment, suggesting that flagellin inhibited cell proliferation. Moreover, after two and three days of treatment, the percentage of BrdUrd-positive cells was further decreased and maintained at 13% 98.

In conclusion, probiotics play a multifaceted role in modulating TLR signaling pathways, which has implications for cancer prevention and therapy. By influencing the immune response through anti-inflammatory cytokines and directly interacting with TLRs, probiotics offer a promising strategy for additional therapeutic interventions targeting cancer progression.

### Probiotics: immune enhancers and anti-proliferative effects

The exploration of probiotics as immune adjuvants has shown some promising findings. To begin with, oral administration of FM with* L. helveticus R389* demonstrates an immunoregulatory response in BC-bearing mice, suggesting its potential as an immune adjuvant therapy 99. Mice treated with Selenium nanoparticles (SeNp)-enriched *L. plantarum* not only had elevated IFN-γ and IL-2 levels but also enhanced NK cells activity, illustrating an induced Th1 bias of their immune response. Moreover, SeNp enriched with *L. plantarum* caused more effective antitumor response among 4T1 BC mice model which showed a reduced tumor growth along with increased survival rate 100.

Similar encouraging results were seen with the oral administration of selenium SeNP-enriched with *Lactobacillus brevis*, which lead to a better disease prognosis among highly metastatic BC-bearing mice 101. Yazdi *et al*. showed that the oral administration of only *L. brevis* did enhance the survival rate of 4T1 tumor bearing mice over a 75 days period; however, this enhancement was not at the same rate observed when mice were treated with SeNP-enriched *L. brevis* (230 days) 101. In the same study, the oral administration of SeNP-enriched *L. brevis* increased the level of IFN-γ which may be the reason for the significant enhancement of NK cell activity 101.

Other research has demonstrated an additional impact of probiotics, highlighting their anti-proliferative effects, primarily through the induction of apoptosis. Experiments were carried out using *L. plantarum*, a component of the gut microbiota. The findings revealed that *L. plantarum* - Conjugated Linoleic Acid (LP-CLA) led to a notable elevation in the pro-apoptotic protein Bax within MDA-MB-231 cells 102. In fact, MDA-MB-231 cells are aggressive, hormone-independent BC cells 103, referred to as "triple-negative" BC, due to the lack of ER, progesterone receptor, or HER2 protein. In order to confirm the induction of apoptosis by LP-CLA treatment in MDA-MB-231 cell lines (human cells), DNA fragmentation analysis and Flow cytometric analysis were performed. The data showed nuclear fragmentation of MDA-MB-231 cells in a dose-dependent manner after 48 h of incubation. These research results reveal that the anti-proliferative effect of LP-CLA against MDA-MB-231 cells was primarily through the induction of apoptosis 102.

## CLINICAL APPLICATIONS OF PROBIOTICS IN BREAST CANCER

As it can be noticed, many *in vivo* and *in vitro* studies have provided evidence that probiotics display activity against BC development and BC prevention. However, scarce number of clinical trials have been done on BC patients. For instance, a case-control study in Japanese population investigated 306 cases with BC and 662 controls aged 40 to 55 years. They investigated their diet, lifestyle, and other BC risk factors using a self-administered questionnaire and interview. The results showed that regular consumption of *L. casei* Shirota and soy isoflavones since adolescence is significantly associated with decreased BC risk in Japanese women 104.

The clinicaltrial.gov web page has registered clinical trials investigating the effect or probiotics in BC. The trial, NCT03290651, included 20 women at high risk for BC and 20 healthy controls. Case patients were randomized (10 per group) to receive either a probiotic (one capsule containing 2.5 billion CFU of *L*. *rhamnosus* GR-1 and *L. reuteri* RC-14) or a placebo, administered once daily for 90 days. The investigators hypothesize that women at risk of BC and women with BC share the same profile of breast microbiome and that oral administration of probiotic *Lactobacilli* can reset this to one found in healthy women. Another ongoing trial, NCT06039644, tends to evaluate the efficacy of probiotics in improvement and prevention of chemotherapy associated side effects in patients with BC.

In addition, it is interesting to clinically see the effects of probiotics in reducing the adverse reactions of chemotherapy treatments. A recent case-control study included a 57-year-old postmenopausal female with BC undergoing adriamycin-cyclophosphamide and taxol-cyclophosphamide chemotherapies for invasive ductalcarcinoma 21. She used nutritional interventions and supplementation instead of loperamide for preventing chemotherapy-induced diarrhea and maintaining gut health. The pre- and probiotics she took included *L. rhamnosus *and *Saccharomyces cerevisiae boulardii *CNCM I-745*, Bifidobacterium lactis *HN019*, L. rhamnosus *HN001*, L. acidophilus *NCFM* and B. lactis *Bi-07. The results showed a *Proteobacteria* abundance of 2.319% (a 44.58% increase), *Bifidobacterium* abundance of 2.719% (a 457.17% increase), the nine analyzed butyrate producers at 15.40% (a 54.83% decrease), and alpha-diversity at 2.58% (a 20.86% decrease). Upon reassessment of GI function, she reported no symptoms of irritable bowel syndrome, diarrhea, or constipation 21.

Technically, there is just a few already executed or running clinical trials investigating the probiotics and effects of in BC patients. However, no clear prospective studies on the actual efficacy of probiotics in the BC prevention or (combinatorial) treatment have been planned yet, prompting the need for condensed and long-term investigations.

## DIETARY IMPACT ON BREAST CANCER

The synergetic relation between the gut and the dietary habits significantly influences the overall well-being. It became remarkable how our dietary preferences exert hefty consequences over the balance of our microbiome. At the forefront of scientific inquiry lies the tangled connection between diet and cancer, where ongoing research unveils a dual role. On one hand, certain food choices might contribute to the onset of cancer, meanwhile investigations also delve into how some dietary patterns show promise in cancer prevention. This nuanced understanding enlightens the importance of evidence-based dietary recommendations for an inclusive cancer risk management.

The analysis of dietary habits in BC patients unveiled exceptional connections between the specific components of the Healthy Eating Index 2015 (HEI2015) and presence of *Acidaminococus, Tyzzerella,* and *Hungatella* genera in the gut microbiome, such as vegetables and dairy for *Hungatella* and whole fruits for *Acidaminococus*. These results imply a potential association between dietary patterns and the composition of the gut microbiome, suggesting potential implications for BC risk and management 105. On the other hand, an increased consumption of ultra-processed food (UPF) proved to have a significant association with increased risk of BC 106. In contemporary society, there is a prevalent shift towards increased consumption of fast food, especially UPF, with a notable departure from traditional and healthful dietary choices. It is helpful to note that UPF are formulations of ingredients, mostly of exclusive industrial use, that result from a series of industrial processes (hence ‘ultra-processed’) 106.

Of note, strict vegetarians have increased fecal excretion of conjugated estrogens compared with non-vegetarians 107. It means that less estrogen is reabsorbed into the bloodstream, leading to lower plasma estrogen levels. This mechanism could have implications for hormone regulation and health, including potential effects on conditions influenced by estrogen levels, such as BC risk, posing potential protective avenues.

### Mediterranean diet against breast cancer

The traditional MD has its origin in the countries of the Mediterranean basin 108, where the sunny and mild climate favors the production of a lot of fruit and vegetables throughout the year 109. Increased consumption of plant-based food (vegetables, fruits, salad), Extra Virgin Olive Oil as the main source of fat, and minimal consumption of red meat, saturated fatty acids and calorie intake, showed an increased microbiota diversity, along with some *Lactobacilli, Roseburia, Rominococci, Bifidobacteri *110. On the other hand, hydroxytyrosol derived from olive fruit decreased the level of *Proteobacteria *and *Firmicutes* 111.

As per findings from a cohort study in the Netherlands involving postmenopausal women, the results based on 2321 cases of incident BC revealed a noteworthy inverse connection with a MD score that excludes alcohol, specifically in cases of ER-negative BC 112. On the other hand, the relationships with total BC and ER-positive BC were relatively weak and did not reach statistical significance 112. In another study conducted in Saudi Arabia, examining a total of 432 female participants (214 cancer cases and 218 controls), showed a protective role for food choices. Only Saudi postmenopausal women who were newly diagnosed with BC and aged above 45 years old were included. The consumption of one to two servings of dairy products per day was shown to be preventive against BC (p= 0.032), as was the consumption of three to five servings of dairy products daily (p= 0.005). Results also showed a preventive effect of consuming one to two servings of legumes per week (p < 0.001), three to five servings of fruits and vegetables per day (p= 0.007), one to two servings of fish and sea food per week (p= 0.001) as well as drinking one to two cups of tea daily (p= 0.002) 113.

In essence, these findings tightly join our dietary choices, the microbial communities, and the tricky signaling pathways within mammary tissue. The MD emerges as a flare of health, by promoting overall well-being and unveiling a potential shield against BC. Delving into the Diet-Microbiota-Cancer interaction, these findings illuminate a path towards deliberated preventive dietary strategies against BC and enhance the probiotic’s anticarcinogenic properties.

## LIMITATIONS IN FIGHTING BREAST CANCER IN EAST MEDITERRANEAN REGIONS 

In 2020, BC affected 81,900 women in the EMR, accounting for the majority of cancer-related deaths 114. By 2040, it’s projected that BC will claim over one million of lives 114. The main obstacles worsening the scenario of BC in EMR is by far socio-economic, where these countries have faced demographic, socioeconomic, and health status changes during the last decades 115.

The high mortality rate of BC in EMR is attributed to absence of early access to diagnosis and treatment, particularly in countries with lower Human Development Index levels. For instance, a recent study by Tamina *et al*. 116 highlighted that almost a third of the participants showed inadequate attitudes toward health-check while more than half had insufficient BC screening knowledge in Lebanon. In the same study, quite a majority of the participants encountered obstacles to mammography screening which is highly related to the exorbitant costs of mammography which is rarely covered by the government. This gives a real example that the economic burdens, including healthcare costs and lost productivity, further strain efforts to combat the fatal disease.

On the other hand, the socioeconomic changes have led to other negative consequences, such as the introduction of various processed foods and new ingredients into dietary styles across the region 115. Populations in EMR countries are undergoing a nutrition transition, shifting from traditional diets to those higher in fat, refined sugar, and processed foods. This critical shift in dietary patterns and lifestyles has been linked to a rising prevalence of diet-related chronic diseases, including cancer 115. For example, the economic challenges in Lebanon depleted food supplies and increased costs, with prices of many traditional, healthy ingredients increasing by 166% with more than 55% of the population living below the poverty line; consequently, maintaining a MD has become largely unaffordable for many 117. It seems that as a direct result of this shift towards unhealthy food consumption, EMR countries are currently facing a rapid increase in rates of not only BC but other types of cancers and diseases.

In addition to the influence of dietary choices, it is crucial to mention other risk factors such as the role of smoking habits in cancer manifestation. So far, cigarette smoking has been identified as a significant risk factor for the development of BC. As reported, BC risk showed a linear increase with the increased intensity and duration of smoking 118. Similarly proved in another study from 2022, smoking increased the risk of BC in the female population, especially premenopausal BC, and this risk was positively associated with the duration and intensity of smoking 119. In the EMR, tobacco consumption among females, especially among school girls, is rapidly rising, reaching over 20% in most of the EMR countries 120. Despite being abundant in probiotic-rich MD food (**Table 4**), the EMR faces other factors such as the ones previously mentioned, forming a large limitation against the MD and probiotics contribution to the BC prevention in EMR.

**Table 4:** Probiotics in some of the common East Mediterranean dishes.

**Food**	**Main Constituents**	**Probiotic present**	**Reference**
**Kishk**	Fermented bulgur and yogurt/milk	Lactic Acid Bacteria	121
**Shanklish**	Aged cheese made from yogurt or milk	Lactic Acid Bacteria	122
**Makdous**	Stuffed eggplants fermented in olive oil	*Lactobacillus* and *Pediococcus*	124
**Ayran**	Diluted yogurt with added salt	*Lactobacillus* and *Streptococcus*	124
**Labne**	Strained yogurt	*Lactobacillus* and *Streptococcus*	124
**Torshi**	Pickled vegetables (e.g., turnips, cucumbers)	Lactic Acid Bacteria	125
**Laban Immo**	Yogurt-based dish with meat and rice	*Lactobacillus bulgaricus*, *Streptococcus thermophilus*, *Lactobacillus acidophilus*	126
**Kefir**	Fermented milk made with kefir grains	*Lactobacillus*, *Bifidobacterium*, *Saccharomyces kefir*	127
**Fattoush Salad**	Mixed salad	*Lactobacillus* and *Pediococcus*	128
**Lebanese Sauerkraut**	Fermented Cabbage	*Lactobacillus plantarum*	129

Nonetheless, diets alone may not be able to overcome environmental factors. Exposure to pollutants, like toxic chemicals, plastics, pesticides and smoke among others, have been shown to modulate hormonal balance, altering normal physiologic responses. Therefore, organizing programs to educate people about the environmental and general risk factors and to dive deeper into research on how probiotics might impact the development of BC could be a start to prevent it.

## CONCLUSION

In summary, probiotics have had significant outcomes *in vivo* and *in vitro* with few clinical applications that need to expand. This review focused on the rising advantages of probiotics, such as anticarcinogenic and antioxidant properties they exert. Probiot-ics offer a promising strategy at the level of BC, along with using the effect of our dietary behaviors on shaping the abundance of these probiotics. We can lay the groundwork of preventive strategies against BC. Hence, more clinical trials and longitudinal studies should be conducted to confirm the probiotics and nutritive pathways as an additional treatment.

As presented, despite the high prevalence of BC in EMR, we can monitor the scarcity of researches in this field and lack of awareness and attention to the issue. This ignorance stems from limited public education on early detection, cultural barriers that discourage open discussions about women’s health, and a healthcare system that may not provide equal access to screening or treatment for everyone. It is clear that "man is averse to what he is ignorant of" so we need widespread public education on BC and the importance of regular BC screen-ing. Future studies should probe into harnessing the MD in the fight against BC in EMR.

## AUTHOR CONTRIBUTION

The idea of the article was provided by [ESA]. Literature search and data collection were performed by [KA], and [ESA], and the drafted article was critically revised by [LF], [AM], and [ESA]. The first draft of the manuscript was written by [KA] and all authors commented on previous ver-sions of the manuscript. The final version was edited by [ESA]. All authors read and approved the final manuscript.

## CONFLICT OF INTEREST

The authors declare no competing interests.
